# Impact of Plant-Based Foods and Nutraceuticals on *Toxoplasma gondii* Cysts: Nutritional Therapy as a Viable Approach for Managing Chronic Brain Toxoplasmosis

**DOI:** 10.3389/fnut.2022.827286

**Published:** 2022-02-25

**Authors:** Sijie Tan, Wen Han Tong, Ajai Vyas

**Affiliations:** School of Biological Sciences, Nanyang Technological University, Singapore, Singapore

**Keywords:** brain, CNS, cyst, chronic toxoplasmosis, nutrition, intervention, parasite, *Toxoplasma gondii*

## Abstract

*Toxoplasma gondii* is an obligate intracellular parasite that mainly infects warm-blooded animals including humans. *T. gondii* can encyst and persist chronically in the brain, leading to a broad spectrum of neurological sequelae. Despite the associated health threats, no clinical drug is currently available to eliminate *T. gondii* cysts. In a continuous effort to uncover novel therapeutic agents for these cysts, the potential of nutritional products has been explored. Herein, we describe findings from *in vitro* and *in vivo* studies that support the efficacy of plant-based foods and nutraceuticals against brain cyst burden and cerebral pathologies associated with chronic toxoplasmosis. Finally, we discuss strategies to increase the translatability of preclinical studies and nutritional products to address whether nutritional therapy can be beneficial for coping with chronic *T. gondii* infections in humans.

## Establishment of Chronic Toxoplasmosis in the Brain

*Toxoplasma gondii* (*T. gondii*) is an obligate protozoan that infects mostly warm-blooded species including humans. Toxoplasmosis affects approximately a third of the human population. *T. gondii* has a few major clonal lineages, namely I, II, and III, and atypical genotypes of differing virulence and epidemiology (1). For example, the type II lineage is predominant in Europe and North America, and higher genetic diversity is observed in South America (2). *T. gondii* infection primarily occurs through the ingestion of oocysts or tissue cysts (3). Mother-to-foetus transmission and transmission through organ transplantation have also been observed (4, 5).

**Figure 1:**
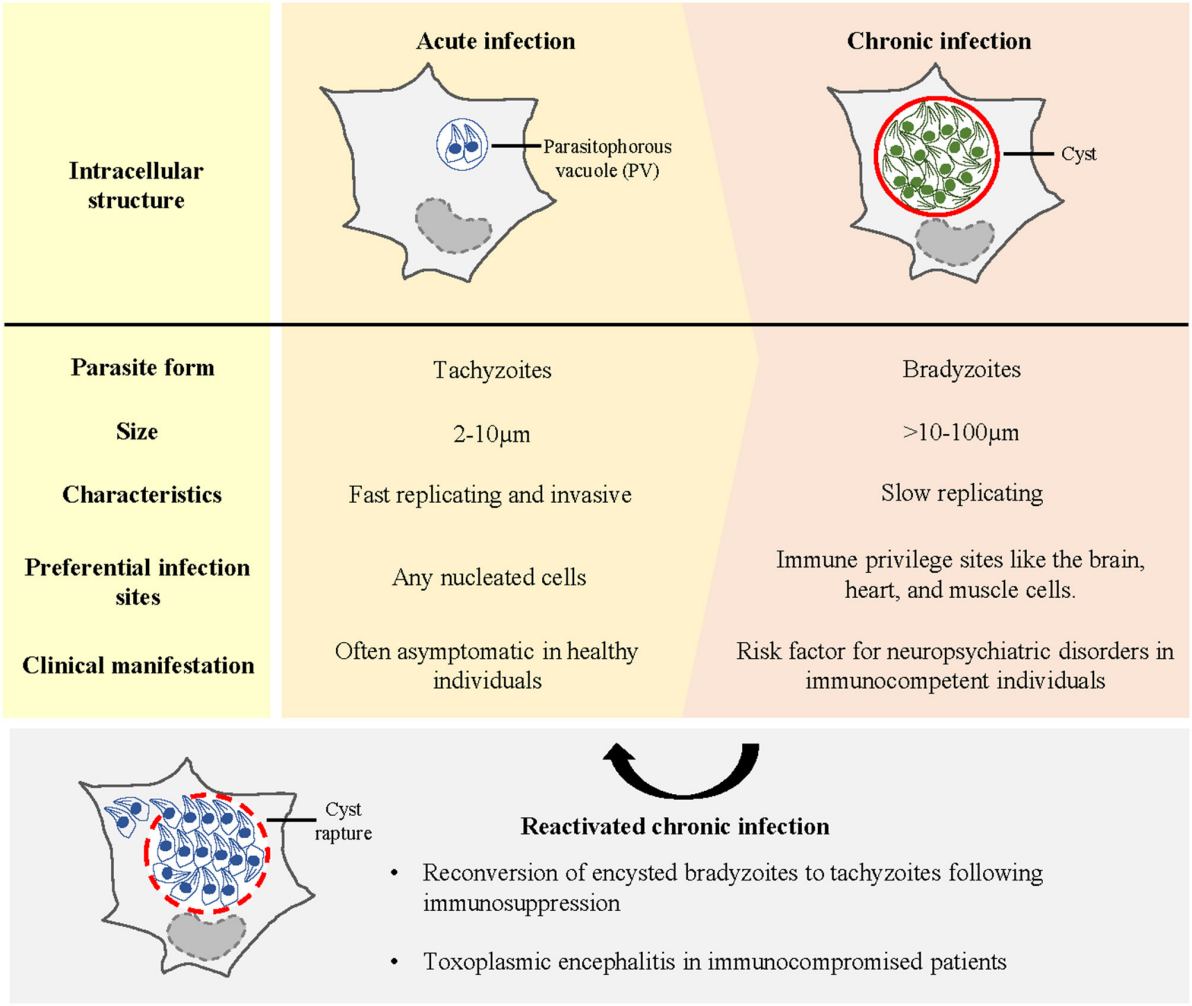
Different stages of *T. gondii* infection in the host. Early or acute infection is characterised by the invasion and intracellular proliferation of tachyzoites in the parasitophorous vacuole (PV). Acute infection is often asymptomatic in healthy individuals. To establish life-long infection, *T. gondii* tachyzoites differentiate into bradyzoites within a cyst. Immune-privileged sites such as the brain provide a niche for *T. gondii* encystment. Chronic infection in the brain has been linked to the incidences of neuropsychiatric conditions. Upon immune suppression, encysted bradyzoites reconvert into tachyzoites, causing toxoplasmic encephalitis in immunocompromised patients.

Following the ingestion of oocysts or cysts, the parasites invade intestinal epithelial cells and transform into tachyzoites. These tachyzoites replicate rapidly inside the parasitophorous vacuole (PV) (Figure 1) and ultimately rupture the PV membrane to infect the neighbouring cells (6). Tachyzoites are disseminated from the intestine to distant tissues by infecting and hijacking the migratory potential of immune cells (7–13). To reach the brain parenchyma, the parasites shuttle across the blood–brain barrier (BBB) within the immune cells or via transmigration of free tachyzoites across endothelial cells (7, 14–17). Unlike peripheral organs where the parasite presence is transient due to the ensuing immune responses [see reviews (18–20)], immune privilege sites such as the central nervous system (CNS) or the brain offer an environmental niche for the parasites (in this review, CNS and brain will be used interchangeably, with the brain being the main point of reference when CNS is used). Once inside the brain, the tachyzoites differentiate into slow-dividing bradyzoites within a cyst (Figure 1). A mature brain cyst (diameter: 50–70 μm) contains up to thousands of bradyzoites (21). Encysted bradyzoites are impervious to the host's immune system and antimicrobial drugs. Hence, *T. gondii* cysts can persist in the brain for a long time and establish a chronic infection. Neurons are the preferred cell type for *T. gondii* encystment, as observed in rodent models (22–24). The interaction between *T. gondii* cysts and human brain resident cells remains elusive. However, the limited data from autopsies have shown that cysts are present in neurons and astrocytes (25). In addition to exhibiting tropism for neurons, *T. gondii* encystment exhibits a high predilection to specific brain areas such as the amygdala, cerebral cortex, hippocampus, and cerebellum in rodents (26–30). The neuroanatomical distribution of cysts in humans is mainly evidenced through the autopsies of acquired immunodeficiency syndrome (AIDS) patients with toxoplasmic encephalitis (TE). TE lesions, which are an indication of cysts' presence and rupture, have been reported in the cerebral cortex, basal ganglia, and cerebellum (31–35).

The strong tropism and persistence of *T. gondii* cysts in the brain confirm that CNS toxoplasmosis impairs the complex brain functioning. Human infection produces a gamut of clinical manifestations, ranging from long-term behavioural and cognitive alterations, to mortality following reactivation of encysted bradyzoites upon immunosuppression (36).

## Clinical Manifestations of Chronic Toxoplasmosis and Its Reactivation in the Brain

Toxoplasmosis is an opportunistic infection in immunocompromised individuals. In reactivated toxoplasmosis, encysted bradyzoites transform back into tachyzoites and proliferate uncontrollably in the brain (Figure 1), causing widespread inflammation and critical morbidity. Reactivated toxoplasmosis develops in patients, such as those with human immunodeficiency virus (HIV)/AIDS, allogeneic haematopoietic stem cell transplant (allo-HSCT) and solid organ transplant (SOT) recipients (5, 37–39), because their T-cell repertoire is perturbed which weakens the host's antiparasitic responses. In HIV/AIDS patients, especially in those with a low CD4^+^ T-cell titre (<200/ml), reactivation of *T. gondii* brain cysts often manifests as TE (40). In allo-HSCT and SOT recipients, cerebral toxoplasmosis accounts for most post-transplant CNS infections, and TE remains the usual presentation (5, 38). Notably, in all cases, the clinical course of untreated TE is fatal.

Unlike in immunocompromised patients, brain cyst reactivation and TE are rare in immunocompetent adults with latent toxoplasmosis. *T. gondii* cysts persist in the brain for a long time. Latent CNS toxoplasmosis manifests asymptomatically in immunocompetent adults because cysts are largely quiescent. However, growing evidence shows that latent infection negatively affects neurocognitive functions. In intermediate rodent hosts, chronic infection is associated with behavioural changes such as reduced anxiety-like behaviour, neophobia, or more specifically, the fear of feline predators (26, 41, 42). Similar sequelae were also observed in infected humans. Latent CNS toxoplasmosis is consistently linked to schizophrenia (43–46). Various meta-analyses have also reported its probable association with anxiety disorders (47), bipolar disorder (43, 48), depression (49), epilepsy (50), and obsessive-compulsive disorder (51), with all these associations relying on the correlative data between *T. gondii* IgG seropositivity (a proxy for chronic infection) and disease incidence. The causal link between latent CNS toxoplasmosis and neuropsychiatric disorders in humans remains unclear. However, preclinical studies have suggested that neurological sequelae are triggered through the modulation of the brain and immune milieu. Detailing the neurobiology underlying the association between latent *T. gondii* infection and neuropsychiatric conditions is beyond the scope of this review [see reviews (52–55)]. However, this neurobiology can be explained by the influence of the parasite or cyst on: (1) specific brain regions for cognition, mood, and emotion processing; (2) neurotransmitter levels (56–61); (3) neuroinflammation (62, 63); and (4) neuroendocrine changes (64–66).

Chronic CNS toxoplasmosis has far-reaching consequences in humans. In immunocompetent individuals, *T. gondii* cysts establish a persistent brain infection that is linked to the incidence of neuropsychiatric disorders. Additionally, immunocompromised patients experience recrudescent latent infection. Given these dire consequences, treatments for preventing the formation or elimination of *T. gondii* cysts from the brain are urgently required to reduce public health burden.

## Treatments for Toxoplasmosis: Current Options and Challenges in a Snapshot

**Figure 2:**
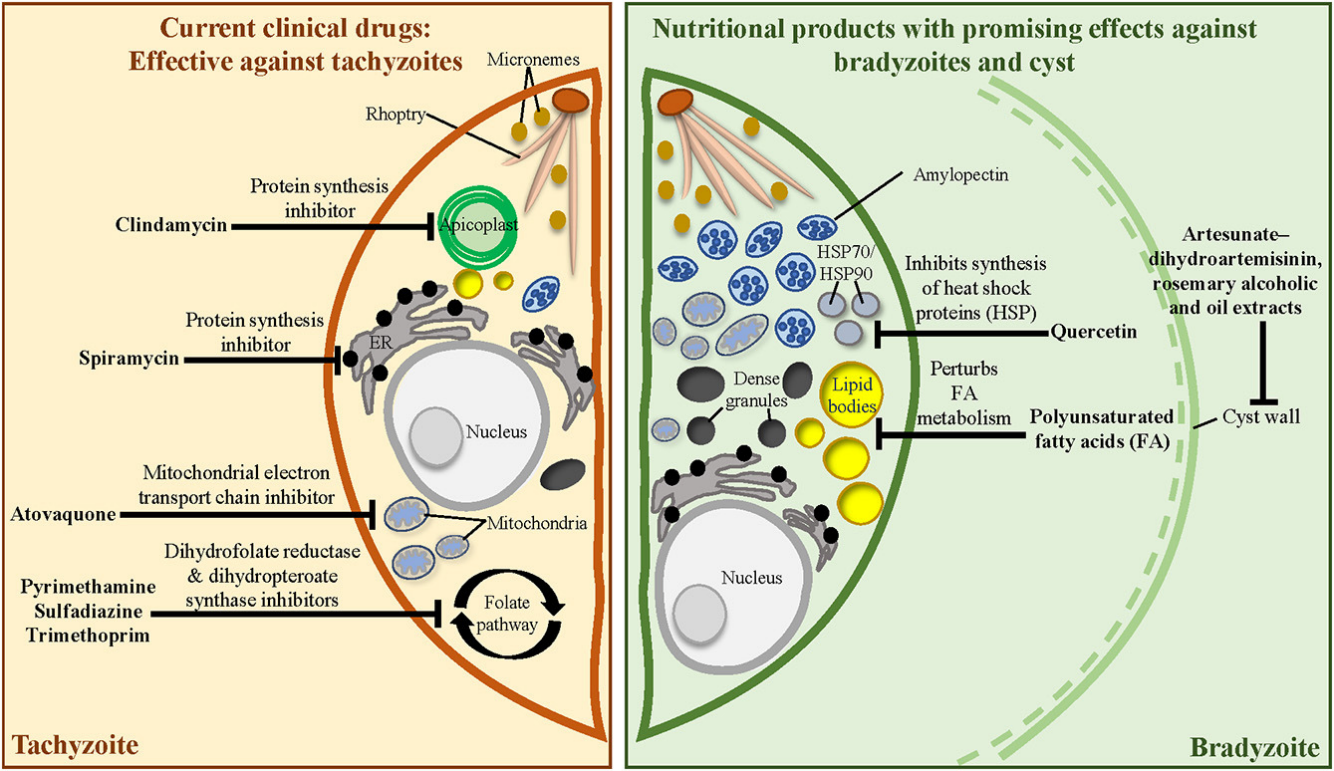
Mechanistic targets of clinically approved anti-*T. gondii* drugs and the known targets of medicinal herbs, phytochemicals, and essential nutrients. (Left) Current clinical drugs target metabolic pathways in the tachyzoite. (Right) Nutritional products may alter bradyzoite biology and cyst wall integrity to alleviate cyst burden during chronic infection.

The exponential rise in knowledge regarding *T. gondii* biology has proven beneficial to uncover new druggable targets for combating toxoplasmosis (67). Several prophylactic and therapeutic regimens have been adopted clinically [see reviews (68, 69)] (Figure 2, left). The standard treatment for toxoplasmosis involves a combination of pyrimethamine (Pyr) and sulfadiazine to inhibit the biosynthesis of folic acids (70)—the precursors for nucleic acid and protein synthesis in *T. gondii* tachyzoites. Folinic acid is often administered concurrently to minimise Pyr-induced haematologic toxicity (71). However, the Pyr–sulfadiazine treatment is associated with multiple adverse side effects, such as poor tolerability (72, 73), and emergence of *T. gondii* strains resistant to Pyr-based therapies (74). Other Pyr-based regimens such as the combination of Pyr with clindamycin (a protein synthesis inhibitor) or atovaquone (a mitochondrial inhibitor) have been adopted in patients with sulpha drug allergy (75, 76). Another regimen involving the combination of trimethoprim (an antagonist of folic acid synthesis) and sulfamethoxazole is also recommended (73). However, this drug combination is only adopted in lower-income countries where Pyr is unaffordable (77). Although other therapeutic options are available, none have been found to be more effective than Pyr–sulfadiazine.

Unfortunately, all currently available therapeutic regimens are only effective against tachyzoites and cannot eliminate tissue cysts. Rational development of new and safe therapeutic interventions against chronic toxoplasmosis is urgently required.

## Impact of Plant-Based Foods, Phytochemicals, and Nutrients on Chronic Brain Toxoplasmosis

The lack of clinical drugs that eliminate *T. gondii* cysts from the brain has driven the search for effective therapeutics. At least 50 promising anti-cyst candidates have been identified through screening of compound libraries, target-based drug design, or repurposing of Food and Drug Administration (FDA)-approved drugs [see review (78)]. For example, imiquimod has recently been identified as a promising prophylactic and therapeutic drug against brain cyst burden and cyst reactivation through its immunomodulatory role (79). While the discovery of pharmacological cysticidal agents is underway, nutritional therapy has emerged as a new approach to cope with chronic *T. gondii* infection. Nutritional therapy is based on the idea that the food we eat can influence health and disease development. This idea has evolved from ancient times when pharmaceutical drugs were unavailable. Despite the advancement of modern medicine, food ingredients are being investigated for their therapeutic potential.

In this review, we evaluate the contribution of nutritional products—extracts from food plants, phytochemicals, vitamins, and minerals—towards the prevention (prophylactic) or treatment (therapeutic) of chronic brain toxoplasmosis (Table 1). These products robustly impacted the brain cyst burden and neurological sequelae in preclinical models of chronic toxoplasmosis. Interestingly, most of the anti-cyst nutritional products also interfere with the tachyzoite stage. In the first part, we highlight the nutritional products without a disease-related target (section Nutritional Products Without Identified Targets). In the next section, we discuss a small pool of nutritional products that targets the bradyzoite biology to interfere with brain cyst development (section Nutritional Products With Identified Targets: Interfering With Bradyzoite Biology and Cyst Development) (Figure 2, right).

**Table 1:** Herbal extracts, phytochemicals, and nutrients effective against *T. gondii* cysts.

**Food plant**	**Bioactive component**	**Animal model**	***T. gondii*** **inoculum**	**Treatment paradigm**	**Efficacy compared to vehicle-treated control group**	**Toxicology findings**	**References**
*Artemisia annua* (Sweet Woodworm)	Artemisinin derivatives: Artesunate–dihydroartemisinin	OF1 mice	DUR cysts	100 mg, 3 times a day for 5 days, after 3 months post-infection.	- Reduction in brain cyst burden by ~40% with irregular cyst structure.	0.1–2 μg/ml artesunate and dihydroartemisinin alone or in combination showed a lack of toxicity against THP-1 cells.	(80)
	Artemisinin derivative: CPH4-136	CBA/J mice	ME49 cysts	3 mg/kg BW/day for 16 or 32 days, after 5 weeks post-infection.	- Reduction in brain cyst burden by ~40%.	Low cytotoxicity and high therapeutic index of >1,500.	(81)
*Curcuma longa* (Turmeric)	Curcumin (Native compound and nanoemulsion)	BALB/c mice	Tehran cysts	100 mg/kg BW/day for 30 days, after 4 h post-infection.	- Reduced number and size of brain cysts: Nanoemulsion is more effective than native compound. - Downregulation of BAG1 expression.	Oral toxicity studies found no clinical symptoms in infected mice administered with the nanoemulsion.	(82)
	Curcumin (Native compound and nanocomposite)	Swiss albino rats	ME49 cysts	10 days, after 60 days post-infection.	- Reduction in brain cysts by 60%: UiO-66-NH_2_ nanocomposite is the most effective.	- N.A.	(83)
*Myristica fragrans* (Nutmeg)	Ethanolic extract from dried fruits	Swiss albino mice	ME49 cysts	500 mg/kg BW/day for 14 days, after 4 days post-infection.	- Mild reduction in number of brain cysts by ~0.8%.	Low cytotoxicity against Vero cells with IC50 at 24.83 μg/ml.	(84)
*Nigella sativa* (Black cumin)	Black seed oil	Swiss albino mice	ME49 cysts	Prophylactic (“P”): 5 ml/kg BW/day for 14 days followed by infection. Therapeutic (“T”): 5 ml/kg BW/day for 14 days, after 4 days post-infection.	- Higher survival rate: 100% for “P”; 86.7% for “T.” - Reduced brain cyst load. - Lessened meningitis, encephalitis, and perivascular cuffing. - “P” was more effective than “T” regimen.	No mortality or clinically significant toxicity in the form of decreased activity, piloerection, lethargy, or weight loss were observed in uninfected mice administered with the black seed oil.	(86)
*Rosmarinus officinalis* (Rosemary)	Alcoholic and oil extracts from leaves	Swiss albino mice	ME49 cysts	Prophylactic (“P”): 400 mg/kg BW/day of each extract for 7 days followed by infection. Therapeutic (“T”): 400 mg/kg BW/day of each extract for 14 days, on the second week post-infection.	- Reduced brain cyst burden, BAG1 expression, and cyst viability: Cysts isolated from “P” and “T” groups displayed mutilation in the surface membrane and were less infective. - Lessened histopathological insults in the brain. - “T” was more effective than “P” regimen.	No significant toxicity observed (87).	(88)
*Salvia miltiorrhiza* (Red sage)	Tanshinone IIA	*In vitro* Vero cells	PLK/DLULC_1C tachyzoites followed by *in vitro* differentiation	1, 2.5, 5, 10 μM for 48 h.	- Reduction in expression of bradyzoite-specific marker.	10 μM did not significantly affect host cell viability (host cell viability after treatment was at 83%).	(89)
*Thymus broussonetii* Boiss	Oil extract from whole plant	OF1 mice	PRU cysts	20 μg at the point of infection, or 30 min, 5, or 6 days post-infection.	- Absence of intracerebral cysts.	20 μg of essential oil did not show toxicity in mice (90).	(91)
*Thymus vulgaris*	Ethanolic extract from leaves	Swiss albino mice	ME49 cysts	Prophylactic (“P”): 500 mg/kg BW/day for 5 days followed by infection. Therapeutic (“T”): 500 mg/kg BW/day for 10 days, after 42 days post-infection.	- Smaller brain cysts with irregular cyst wall, and reduction in cyst count in “P” (24%) and “T” (46%) groups. - Amelioration of neuroinflammation and neuronal necrosis.	N.A.	(84, 92)
	Ethanolic extract from aerial parts	Swiss albino mice	ME49 cysts	400 mg/kg BW/day for 14 days, after 4 days post-infection.	- Reduction in brain cysts by ~47.5% and lessened inflammatory brain lesions.	N.A.	
Fruits, vegetables, and grains	Quercetin	*In vitro* human fibroblast cells	ME49 tachyzoites followed by *in vitro* differentiation	100 mM.	- Reduced bradyzoite antigen-positive *T. gondii* vacuoles.	N.A.	(93)
Pomegranate	Urolithin-A	BALB/c mice	PRU tachyzoites	30 μg for 5 weeks, after 2 days post-infection.	- Reduced brain cyst size. - Less anxiolytic behaviour.	50 and 100 μM did not elicit toxicity against neuronal cells.	(94)
Berries, grapes, red wine	Resveratrol (Native compound or nanoparticles)	Swiss albino and BALB/c mice	VEG cysts	100 mg/kg BW with 0.5 mg/kg BW sulfamethoxazole–trimethoprim for 10 days, after 20 days post-infection.	- Reduced number of brain cysts. - Improved memory and heightened anxiety behaviour.	N.A.	(95–97)
Fish, vegetable-, and nut-based oil	Omega-3 polysaturated fatty acids	Fat-1 transgenic mice	ME49 cysts	–	- Reduced number of brain cysts.	N.A.	(98)
		Kunming mice	RH tachyzoites	1 g/kg BW fish oil for 4 days followed by infection.	- Mice did not develop toxoplasmosis.	N.A.	(99)
Multiple food sources	Selenium and vitamin E	Swiss webster and C57BL/6 mice	ME49 cysts	Mice were placed on a diet deficient in selenium and vitamin E for 3 weeks, followed by infection.	- Reduced number of cysts and little evidence of neuroinflammation.	N.A.	(100)
Multiple food sources	Copper, selenium, and zinc (In biogenic forms)	BALB/c mice	Tehran cysts	Cu: 2, 4 mg/kg Se: 2.5, 5, 10 mg/kg Zn: 32.5, 75, 150 mg/kg 14 days treatment, followed by infection.	- Reduced brain cyst burden and increased anti-inflammatory iNOS expression.	No significant toxicity in mice based on liver and renal enzyme activities, and haematological parameters.	(101–103)

*BW, Body Weight; N.A. means that toxicology findings were not available or not discussed in the studies*.

### Nutritional Products Without Identified Targets

#### Herbal Extracts

Herbs are extensively used for culinary purposes and are a crucial part of the human diet and culture. Additionally, herbal extracts are recognised for their health-promoting effects (104, 105). Different cultural or societal backgrounds (e.g., traditional Chinese medicine and traditional Korean medicine) offer the propriety of herbal products for medicinal use.

*Myristica fragrans* (nutmeg), an aromatic evergreen plant, is extensively used as a food spice and in folk medicine to treat ailments (106). Its bioactive constituent myrislignan guards against acute cerebral toxoplasmosis by perturbing the tachyzoite's mitochondrial function (107, 108). *Myristica fragrans* is also effective against the bradyzoite stage. In mice infected with the type II ME49 *T. gondii* strain, ethanolic extract from dried fruits of *M. fragrans* mildly protected against brain cyst development (84).

*Nigella sativa* (black cumin), another food flavouring agent, has an enduring history of usage in folk medicine (109). Extensive clinical trials have highlighted the safety and efficacy of *N. sativa* in the treatment of inflammatory, auto-immune, and metabolic disorders (110). The volatile oil component of *N. sativa* acts synergistically with the current clinical drug Pyr to reduce *T. gondii* load and pathological insults to the liver and spleen of acutely infected mice (85). *N. sativa* seed oil also has prophylactic and therapeutic effects on chronic infection. In a murine infection model induced with ME49 *T. gondii*, parasite cysts were accumulated in the brain 5 weeks after infection, eventually leading to death of the mice (86). However, *N. sativa* seed oil administered before infection or a few days after infection significantly reduced the mortality and the brain cyst load in infected animals (86). The treated groups also exhibited less brain histopathological alterations compared with the untreated group (86). An enhanced brain level of inducible nitric oxide synthase (iNOS) was suggested to protect the treated animals against immunopathology (86). Interestingly, the prophylactic regimen is more effective than the therapeutic regimen in enhancing resistance to chronic toxoplasmosis (86).

Species of the *Thymus* genus, a group of aromatic herbs used as flavour additives, have been extensively studied for their antimicrobial activities (111, 112). Administering essential oils from *Thymus broussonetii* Boiss at the time of infection or a few days after infection prevented chronic brain toxoplasmosis. Mice infected with the type II Prugniaud (PRU) *T. gondii* strain developed intracerebral cysts after 3 weeks (91). However, animals treated with the oil extract did not exhibit brain cysts (91). In another study, ethanolic extract from the leaves and ariel parts of *Thymus vulgaris* reduced the severity of chronic infection. Administration of the herbal extract prophylactically to chronically infected mice has been shown to reduce the brain cyst load and ameliorate the inflammatory brain lesions (84, 92).

#### Phytochemicals

Polyphenols are produced by plants to combat environmental insults (113). In humans, dietary intake of fruits and vegetables rich in these phytochemicals promotes preventive and therapeutic benefits against various disorders such as cancer, cardiovascular diseases, chronic degenerative diseases, and metabolic syndromes (114). In addition to their medicinal application, these natural compounds are being targeted by the blooming nanotechnology industry. With nanotechnology, the stability, delivery, and release of dietary polyphenols to the brain tissue have markedly improved, greatly enhancing the clinical translatability of these compounds to treat CNS diseases (115).

The rhizome of *Curcuma longa* (turmeric), a spice best known for its use in curry, exhibits therapeutic potential against various diseases. Its potential can be attributed to the presence of curcumin, a highly pleiotropic compound that targets multiple biological signalling pathways (116). For example, curcumin targets the *T. gondii* detoxification pathway and blocks parasite growth by inhibiting *T. gondii* glyoxalase 1 activity (117). Extensive clinical trials have highlighted the safety and efficacy of curcumin in treating multiple human diseases (118). Curcumin has been designated as a Generally Recognised as Safe (GRAS) dietary compound by FDA. However, the bioactivity of curcumin is often limited by its low oral bioavailability (119). Nanocurcumin (encapsulation of curcumin in nanomaterials) has improved the solubility and therapeutic applications of curcumin. Curcumin and nanocurcumin have been widely investigated as antiparasitic agents (120). In an experimental model of acute toxoplasmosis, curcumin and curcumin nanoemulsion improved survival and reduced peritoneal tachyzoite load in the mice infected with the virulent type I RH *T. gondii* strain (82). In a chronic experimental paradigm, curcumin and the nanoemulsion administered shortly after infection with type II Tehran *T. gondii* were found to reduce the brain cyst number and cyst size in chronically infected mice (82). However, the nanoemulsion was found to be more effective in alleviating brain cyst burden than the native curcumin (82). Curcumin and a novel curcumin nanocomposite (prepared by reacting curcumin with amino-functionalised metal-organic frameworks) also reduced brain cyst load in chronically infected rats (83); the nanocomposite yielded a better effect than the native compound in alleviating cyst burden (83).

Resveratrol is a polyphenol enriched in multiple common food products such as berries, grapes, and peanuts. Several *in vitro* and *in vivo* studies have shown that similar to curcumin, resveratrol targets multiple cellular signalling molecules and can affect the initiation and progression of human diseases (121). The efficacy, safety, and pharmacokinetics of resveratrol have been investigated in numerous clinical trials, with the nutraceutical exhibiting promising therapeutic effects against cancer and neurological, cardiovascular, and metabolic diseases (122, 123). Nanotechnology-based formulations for resveratrol have been developed to enhance its bioactivity and bioavailability. Native resveratrol and the nanoparticles displayed synergy with sulfamethoxazole–trimethoprim (ST) to treat chronic *T. gondii* infection. In mice infected with type III VEG *T. gondii*, resveratrol alone showed no significant effect on the brain cyst burden (95–97); however, when co-administered with ST, resveratrol significantly reduced the number of brain cysts and increased the brain antioxidant levels (95–97). Specifically, the pairing of resveratrol nanoparticles with ST has the greatest effect on alleviating brain cyst burden, decreasing the levels of serum pro-inflammatory cytokines, and increasing the level of interleukin-10 (95). The anti-inflammatory effect could be partly mediated by the reduction in purinergic signalling (124, 125), which is a pro-inflammatory immunological pathway that is upregulated during cerebral infection (126). The resveratrol + ST regimen also mitigated downstream behavioural and cognitive impairments observed during chronic CNS infection. Chronically infected animals administered with resveratrol (free or nanoform) and ST spent less time in the open arms of the elevated plus-maze, displaying heightened anxiety behaviour compared with untreated animals (97). The regimen also led to memory improvement in the infected mice in the passive avoidance task (97). On the other hand, administration of free resveratrol or the nanoparticles or ST alone showed no effect on the neurological sequelae (97).

Pomegranate is a rich source of ellagitannins, a polyphenol class metabolised to urolithins by the human gut microbiota (127). Urolithin-A (UA) is regarded as powerful neuroprotectant *in vivo* against neurodegeneration, ischaemic neuronal injury, and brain ageing (128–135). In a pioneer human clinical trial, UA displayed a favourable safety profile and molecular signature for improved muscle health in elderly individuals (133). Our group recently showed that pomegranate and UA exhibit anti-*T. gondii* activity. Urolithin-A treatment interfered with intracellular tachyzoite growth and cyst formation in infected neural cells (94). *In vivo*, daily injection of UA shortly after PRU *T. gondii* infection reduced the severity of chronic CNS infection, which was evident from the smaller brain cysts in chronically infected mice than those in untreated animals (94). Interestingly, chronic infection reduces innate fear of mice towards predatory odour (26, 41, 42). However, UA attenuated the anxiolytic behaviour in chronically infected mice, as evident from the increased fear of these animals towards predatory odour compared with untreated animals (94).

Tanshinone IIA is a major lipophilic phytochemical isolated from the roots of *Salvia miltiorrhiza* Bunge (red sage). This herbal medicine is widely prescribed to treat cardiovascular and inflammatory diseases (136). In a chemical compound library screening to identify anti-*T. gondii* drugs, tanshinone IIA was considered active against *in vitro* tachyzoites and bradyzoites and thus a promising therapeutic candidate for acute and chronic infections (89). However, its effectiveness *in vivo*, particularly the effect against cerebral cyst burden, remains to be investigated.

#### Vitamins and Minerals

Vitamins and minerals are essential micronutrients present in various foods that coordinate a range of human physiological functions. The consumption of different nutritious foods is the best approach to obtain vital nutrients. Vitamins C, D, and E act against *T. gondii* tachyzoites potentially by activating the NO-mediated mechanism to enhance parasite killing (137–139). However, vitamin E seems to be detrimental to chronic toxoplasmosis development. Dietary administration of vitamin E along with selenium prior to infection increased toxoplasmosis severity in infected mice, as evident from the higher cyst burden and severe meningoencephalitis in the brain tissues of these mice (100). The mechanism through which these micronutrients aggravate the phenotype remains unknown; however, preliminary evidence suggests that vitamin E and selenium perturb the protective cytokine expression (140).

Dietary intake of a small quantity of trace minerals, which play crucial roles at cellular and tissue levels, is important mainly for bodily functions and the immune system. Selenium, zinc, and copper are the trace minerals that influence the development of acute and chronic toxoplasmosis. Studies have explored the impact of biogenic minerals, which exhibit reduced toxicity due to the coating of plant molecules, on toxoplasmosis. Biogenic selenium orally administered to acutely infected mice reduced the peritoneal tachyzoite load and mortality (141). In chronic experiments, early supplementation with biogenic selenium, zinc, or copper prophylactically reduced the number of brain cysts or completely prevented their development (101–103). Additionally, these animals exhibited increased iNOS and reduced pro-inflammatory cytokine levels, which suggest the role of these biogenic minerals in strengthening the cellular immune system and protecting animals against harmful neuroinflammatory responses (101–103).

### Nutritional Products With Identified Targets: Interfering With Bradyzoite Biology and Cyst Development

#### Perturbing Tachyzoite Differentiation

Differentiation of tachyzoites into encysted bradyzoites is critical for the persistence of brain toxoplasmosis. Hence, the strategies that interfere with this transformation can reduce cyst formation. The molecular determinants that underlie this transformation have been extensively investigated. Recent discoveries of transcriptional programmes governing this switch have unveiled new therapeutic targets to cease parasite encystment (142–146).

Heat shock proteins (HSPs) are evolutionarily conserved proteins that are expressed in many organisms under stress (147). In *in vitro* culture, stressors such as an increase in pH and temperature can induce tachyzoite differentiation into bradyzoites (148). During this transformation, *T. gondii* upregulates HSP70 and HSP90 expressions (93, 149). HSP70 expression was also detected in the brain cysts of chronically infected mice (150). The molecular role of HSP during differentiation remains unexplored; however, a study demonstrated that HSP90-deficient parasites could not develop into bradyzoites (149). Quercetin, a ubiquitous flavonoid common in many fruits and vegetables, suppresses tachyzoite differentiation into bradyzoites *in vitro* plausibly by inhibiting *T. gondii* HSP70 and HSP90 synthesis (93). Although whether quercetin could influence the brain cyst burden *in vivo* is unknown, its ability to cross the BBB suggests its potential therapeutic value against chronic CNS toxoplasmosis (151, 152).

#### Interfering With Cyst Wall Integrity

Following tachyzoite differentiation into bradyzoites, the PV membrane encapsulating the tachyzoites matures into the cyst wall (153). The cyst wall is composed of a loose inner layer facing the cyst matrix, a dense middle layer, and an outer membrane layer (154). This wall shields the bradyzoites from immune recognition and destruction. Given its role in survival and persistence of bradyzoites, the cyst wall is an attractive pharmacological target for preventing proper cyst development.

Alcoholic and oil extracts of *Rosmarinus officinalis* (rosemary), a medicinal herb commonly used as a culinary spice, exert remarkable prophylactic and therapeutic effects on chronic toxoplasmosis by perturbing cyst wall integrity. Administration of alcoholic and oil extracts from *R. officinalis* leaves to mice 1 week before infection or 2 weeks following infection with ME49 *T. gondii* significantly reduced the brain cyst burden, cyst viability, and histopathological insults compared with that in the untreated group (88). Ultrastructural analysis of the cyst through scanning electron microscopy revealed abnormal cyst morphology in the brain of the treated animals, with the cyst surface marked with multiple depressions, protrusions, and irregularities (88). Isolation and inoculation of these damaged cysts into naive mice did not produce successful secondary infection, highlighting a loss in cyst viability (88). Overall, *R. officinalis* extracts acted as a stronger therapeutic agent than a prophylactic agent.

Artemisinin, the bioactive constituent of the traditional Chinese herb *Artemisia annua*, and its derivatives represent a powerful class of medicines against malaria (155). The similarity in morphology and biochemistry of *T. gondii* and *Plasmodium* has led to the repurposing of artemisinin and its derivatives for toxoplasmosis treatment (156). Artesunate, artemether, and other novel derivatives have been reported to exhibit better potency than artemisinin against tachyzoite growth *in vitro* (80, 157–159). Artemiside and artemisone are two other derivatives that could control acute infection and protect chronically infected mice from reactivated toxoplasmosis (160). Artemisinin appears to act through multiple targets to control tachyzoite growth, for example, by interfering with the parasite's calcium homeostasis and mitochondrial physiology (161–163). The C-10 carba-linked unsaturated derivative CPH4-136 significantly reduced the brain cyst burden in chronically infected mice (81). In another study, co-administration of artesunate and dihydroartemisinin interfered with chronic toxoplasmosis progression by perturbing cyst wall integrity. Feeding artesunate–dihydroartemisinin to the mice chronically infected with the low-virulence DUR *T. gondii* strain resulted in smaller brain cysts and abnormal cyst morphology characterised by the alteration of inner membranes (80). However, these cysts remained viable and continued to produce secondary infection in mice (80).

#### Interfering With Bradyzoite Metabolic Needs

*T. gondii* is auxotrophic for several essential metabolites and therefore depends on the host cell for nutrients. Bradyzoites can access the host's cytoplasm and scavenge nutrients, even when encysted, to meet their energy and anabolic requirements (164–166). *T. gondii* also modulates host's metabolic pathways during infection, thereby increasing the metabolite abundance to support toxoplasmosis progression (167). Metabolic profiling of the cerebral cortex in infected mice revealed a time-dependent upregulation of the metabolites associated with the unsaturated fatty acid (FA) biosynthesis pathway (168). This hints at the role of unsaturated FA in the pathogenesis and progression of cerebral toxoplasmosis. *T. gondii* bradyzoites scavenge and sequester the host's unsaturated FA as an energy source, a trait seemingly critical for proper cyst development (164). Strategies that change the host's reservoir of unsaturated FA could influence cyst development and the progression of chronic toxoplasmosis.

Lipids are a crucial energy source and structural element for cell membranes. Humans can synthesise almost all types of lipids except for unsaturated FA, which they need to acquire from diets including fish, vegetables, and nut-based oils (169). A few studies have highlighted the *in vitro* and *in vivo* efficacy of polyunsaturated FA against acute and chronic toxoplasmosis, possibly by inducing overt FA metabolism in *T. gondii*. Unsaturated FA made tachyzoites more vulnerable to lipotoxicity by inducing improper lipid storage in the parasites (164). Feeding mice with fish oil prevented toxoplasmosis potentially by disturbing the host's and parasite's FA metabolism and FA availability for parasite growth (99). Chronically infected transgenic mice with constantly elevated levels of omega-3 polyunsaturated FA exhibited lower brain cyst burden than wild-type mice (98).

## Translating Research Into Clinical Practice: Obstacles and Strategies to Improve Translatability

Preclinical studies have highlighted the impact of nutritional products on the development of chronic CNS toxoplasmosis. However, as with all animal studies, the validity of the preclinical findings for clinical practice remains questionable. Here, we discuss the factors that could limit the translatability of animal studies and nutritional products and highlight potential strategies that could improve translational success.

Most of the aforementioned animal studies are in their pilot or preliminary stage. These studies have typically evaluated the therapeutic efficacy of a nutritional product with a specified administration route, a single or limited number of dosing, and within a predetermined treatment window period. Extensive optimisation and assessment of pharmacokinetics/pharmacokinetics of the nutritional products are required to translate the preclinical efficacy to human subjects. Moreover, chronic infection in animal models adopted in these studies was induced with a single *T. gondii* strain and in a predefined window period. Due to varying virulence across the parasite strains and the different incubation periods for chronic infection depending on the host species, no single animal model can reflect the development and progression of chronic toxoplasmosis in humans. Overall, the lack of extensive knowledge about the pharmacological properties of nutritional products and an appropriate infection model impedes clinical translation. To this end, delineating the ADMET (absorption, distribution, metabolism, excretion, and toxicity) profiles of the nutritional products can aid optimisation for subsequent preclinical therapeutic studies (170). Uncovering mechanistic targets of the nutritional products will also help in assessing the bioavailability, engagement, and expression of pharmacological activity at the target site. To improve the predictive translatability of animal models, the nutritional products must be tested in a large sample and in different infection models. Studies involving immunodeficient animal models (e.g., dexamethasone-induced immunosuppression) could also help in determining whether nutrition mitigates the recrudescence of latent CNS infection (79).

Rigour interrogation of animal studies can improve the translation process for clinical trials. However, the properties of the nutritional products per se could also limit their intrinsic translation value. Oral bioavailability is probably the greatest bottleneck that limits the therapeutic efficacy of nutritional products in humans. While polyphenols have shown an impact on brain cyst burden in animal models (section Phytochemicals), consumed polyphenols such as curcumin, resveratrol, and quercetin have very low oral bioavailability in humans due to extensive metabolism and rapid elimination from the body (171). Moreover, the water-soluble nature of polyphenols can remarkably impede their uptake across the BBB. Therefore, several studies have used nanodelivery systems to circumvent the difficulties associated with poor blood plasma and tissue bioavailability of polyphenols. Consequently, preclinical success has been achieved in improving the efficacy of curcumin and resveratrol to target *T. gondii* cysts in the brain (section Phytochemicals). The design of semisynthetic derivatives represents another strategy to improve bioavailability. Identification of the structural modifications that improve the stability of phytochemicals in the blood plasma can enhance therapeutic effectiveness. Successful examples include artemisinin derivatives, such as FDA-approved artesunate, artemether, and artemisone. These compounds have better plasma stability, low neurotoxicity, and high potency against toxoplasmosis (section Interfering with Cyst Wall Integrity). Overall, the improved blood plasma and tissue concentrations of nutritional compounds indicate that a low dosage may be required for therapy, which in turn may produce an effective treatment with fewer side effects in humans.

Side effects and tolerance are important considerations for the clinical translatability of any treatment. Most animal studies discussed in this review have considered the cell/tissue injury markers and survival rate as endpoints to evaluate nutritional product toxicity. None of the studies have reported significant toxicity of these products to the host cells and animals. Large-scale intervention trials conducted for curcumin, resveratrol, vitamins, and minerals for human diseases have also established the toxicology profiles for these products (118, 122), thereby laying a foundation for determining the dosing regimens of these products for chronic toxoplasmosis management in a straightforward manner.

Although targeting cyst development is an attractive strategy to interfere with the establishment of chronic brain infection, administering therapy solely targeting the bradyzoite stage may incur the risk of overt tachyzoite accumulation in peripheral tissues. While a healthy immune system can clear peripheral tachyzoites, immunocompromised patients may experience consequences of the parasite burden. Hence, the treatment for chronic brain toxoplasmosis should be effective against both tachyzoite and bradyzoite stages. Most nutritional products highlighted in the present review can target both parasite stages, representing a unique pool of products with dual pharmacological activities. Otherwise, an anti-tachyzoite drug needs to be co-administered with an anti-cyst agent to prevent overt tachyzoite accumulation. Exploring whether anti-cyst nutritive products can act synergistically with current clinical drugs (which are effective only against tachyzoites) to eliminate human infection would be interesting.

The absence of clinically appropriate biomarkers for measuring brain cyst burden is probably the most significant impediment in directly assessing the effectiveness of nutritional therapy in humans. Routine diagnosis of toxoplasmosis in humans relies on detection of parasite's DNA in body fluids through serologic testing or molecular techniques (172). Neuroimaging techniques that are particularly relevant to immunocompromised patients with CNS toxoplasmosis, such as magnetic resonance imaging (MRI) and computed tomography (CT), can efficiently determine TE severity based on ring-enhancing brain lesions (39). However, these tests do not provide direct readouts for the brain cyst burden. The lack of non-invasive techniques or practical quantitative biomarkers to monitor bradyzoite development in the brain makes it difficult to evaluate the efficacy of nutritional therapy in humans. This is particularly a problem when monitoring disease progression in immunocompetent patients with subclinical chronic infection. Consequently, nutritional therapy might need to be validated by monitoring clinical manifestations central to chronic CNS toxoplasmosis. In immunocompetent patients, the emerging association between chronic infection and neuropsychiatric conditions suggests that cognitive failure can predict a latent infection. Thus, the effectiveness of nutritional therapy can be evaluated by monitoring the severity of behavioural symptoms over the course of intervention. In schizophrenic patients with presumptive latent toxoplasmosis, two pilot studies have investigated the effect of co-administering artemisinin or artemether with their usual psychiatric medications and determined if the clinical benefits could be augmented by inhibiting brain parasitism (173, 174). Although the regimens were found to be safe and well-tolerated, adjunctive artemisinin or artemether did not alleviate psychotic symptoms or cognitive deficits (173, 174). In immunocompromised patients, MRI or CT can provide information on TE lesions, which are located proximal to the cysts.

## Closing Remarks

Chronic brain toxoplasmosis poses a significant health threat, causing severe neurological manifestations in immunocompetent adults and immunocompromised patients. Despite persistent efforts by the global disease control group, none of the drugs have been found to successfully eliminate *T. gondii* cysts from the brain. During the last few decades, the interest in repurposing nutritional products with a long-standing history of health benefits as medicinal sources for chronic CNS infection has increased. Preclinical studies have highlighted the promising potential of nutritive food products to prevent or interfere with disease progression; however, evidence supporting human clinical intervention is lacking. To improve the translation process, preclinical studies need to be conducted with the same rigour analogous to clinical trials. There is also a need to identify clinically appropriate biomarkers to monitor the progression of CNS toxoplasmosis. Finally, large-scale clinical trials are warranted to validate the relevance of nutritional therapy for managing human infection. Currently, polyphenols are probably the most promising candidates, as they have been investigated in multiple clinical trials for other human diseases.

## Author Contributions

ST and WHT prepared the manuscript. AV reviewed the submitted version. All authors made substantial intellectual contributions to the work and approved the final manuscript.

## Funding

This work was financially supported by the Ministry of Education, Singapore (grant RG136/15) and Human Frontier Science Programme (grant RGP0062/2018).

## Conflict of Interest

The authors declare that the research was conducted in the absence of any commercial or financial relationships that could be construed as a potential conflict of interest.

## Publisher's Note

All claims expressed in this article are solely those of the authors and do not necessarily represent those of their affiliated organizations, or those of the publisher, the editors and the reviewers. Any product that may be evaluated in this article, or claim that may be made by its manufacturer, is not guaranteed or endorsed by the publisher.
